# COVID-19 testing, incidence, and positivity trends among school age children during the academic years 2020–2022 in the State of Qatar: special focus on using CDC indicators for community transmission to evaluate school attendance policies and public health response

**DOI:** 10.1186/s12887-024-04833-9

**Published:** 2024-05-30

**Authors:** Mohamed Ghaith Al-Kuwari, Azza Mustafa Mohammed, Jazeel Abdulmajeed, Hamad Al-Romaihi, Maryam Al-Mass, Shaikha Sami Abushaikha, Soha Albyat, Shazia Nadeem, Mujeeb Chettiyam Kandy

**Affiliations:** 1 Primary Health Care Corporation-Qatar, Corporation, Doha, Qatar; 2 Ministry of Public Health- Qatar, Doha, Qatar; 3 College of Medicine, Qatar University, Doha, Qatar

**Keywords:** COVID-19, Children, Schools, CDC, Epidemiology

## Abstract

**Background:**

There exists a gap in our understanding of the age-dependent epidemiological dynamics of SARS-CoV-2 among school-age children in comparison to adults within the State of Qatar. Additionally, there has been limited assessment of the timely implementation of physical distancing interventions, notably national school closures, and their impact on infection trends.

**Methods:**

We used the national database to capture all records of polymerase-chain-reaction (PCR) testing, and rapid antigen tests (RAT) conducted at all health care venues in Qatar and administered between August 26, 2020, and August 21, 2022, across all age groups (≥ 5 years old). Study participants under 18 years old were categorized into two age brackets: (5–11) and (12–17), aligning with the Primary and Preparatory/Secondary grade levels in Qatar, respectively. We assessed age group testing rates, incidence rates, and positivity rates in relation to adults. These epidemiological metrics were compared with the CDC’s thresholds for COVID-19 community transmission.

**Results:**

Throughout the school years of 2020–2021 and 2021–2022, a total of 5,063,405 and 6,130,531 tests were respectively conducted. In the 2020–2021 school year, 89.6% of the tests were administered to adults, while 13.7% were conducted on children in the following year. The overall test positivity rates for the 2020–2021 and 2021–2022 school years were 5.8% and 8.1%, respectively. Adolescents underwent the fewest tests during the full study period compared to both adults and young children. Using the CDC indicators, we found that children and adolescents can significantly contribute to elevated infection rates, potentially driving community transmission upon relaxation of social restrictions.

**Conclusion:**

It is crucial to acknowledge the potential for higher transmission among youth and adolescents when formulating transmission control strategies and making decisions regarding school closures. Employing data-driven indicators and thresholds to monitor COVID-19 community levels is important for informing decision-making. These approaches also enable the prompt implementation of infection control transmission mitigation measures in future pandemics.

**Supplementary Information:**

The online version contains supplementary material available at 10.1186/s12887-024-04833-9.

## Background

The State of Qatar had 514,524 cumulative cases of the novel coronavirus disease (COVID-19), resulting in more than 600 deaths as of January 2024 [1, 2]. The country ranked among the top five nations in the East Mediterranean Region with the highest attack rate per 100,000 population [2]. Nakhaeizadeh and colleagues reported that Qatar ranked the first based on COVID-19 infection rates in the region followed by Bahrain, Kuwait, and United Arab Emirates [3]. Testing within the healthcare system in Qatar is conducted on a large scale, primarily for routine purposes, with approximately 5% of the population undergoing testing every week [4]. The first epidemic wave in Qatar was predominantly driven by the original SARS-CoV-2 virus [4, 5]. The initial recorded cluster of community transmission was identified on March 6, 2020 [4, 5]. In response, Qatar adopted public health measures including extensive testing, contact tracing, quarantine as well as physical distancing strategies and schools’ closure to slow the pandemic and reduce the incidence of new cases. Consequently, all schools in the country were closed on March 10th of 2020. Face-to-face classes were suspended, and students continued their learning through online learning platforms [6].

The Ministry of Education and Higher Education (MOEHE) in Qatar initiated a phased reopening of schools at the end of August 2020, implementing a three-stage back-to-school plan on September 1st [7]. Throughout the 2020–2021 academic year, Qatar adopted a blended learning approach, integrating online instruction with traditional classroom-based education. This strategy incorporated enhanced hygiene protocols such as mandatory mask-wearing and frequent hand washing. Furthermore, schools implemented reduced classroom capacities and student cohorting [6].

The initial phase commenced with on-campus attendance of 50% of students. It was anticipated that by the third phase, students would transition to full-time classroom attendance. Nevertheless, the nation experienced a significant SARS-CoV-2 surge characterized by the predominance of the Alpha (B.1.1.7) and Beta (B.1.351) variants, reaching its peak in the first week of April 2021 [5, 8, 9]. As community cases began to rise by March 2021, schools reduced their on-campus attendance to 30%, transitioning to fully online instruction for all students by April 2021. The Delta (B.1.617.2) variant began to emerge in Qatar by the end of March 2021 [9] (refer to Supplementary files “SF1”, “SF2”).

In the following academic year of 2021–2022, schools initially implemented a distance-learning mode. They swiftly transitioned to 100% face-to-face attendance as the pandemic entered a low-incidence phase, dominated by the Delta variant, from mid to late 2021 [5, 8–10]. As COVID-19 cases began to escalate due to the Omicron variant in December [5, 8], the Ministry opted to prolong the winter holidays, suspending school attendance for all students in both public and private schools throughout January 2022. By February, schools resumed operations at 100% capacity, contingent upon students presenting a weekly pledge form signed by parents as proof of a negative rapid antigen test conducted at home within 48 h prior to school entry, (refer to Supplementary files “SF3”).

There is a gap in our knowledge regarding understanding the age-dependent epidemiological dynamics of SARS-CoV-2 among school age children compared to adults in the State of Qatar. Studies conducted in the earlier months of the pandemic indicated that children in general have lower COVID-19 incidence rates [7]. Moreover, infection trends in multiple countries suggested that school-based transmission was not more prevalent than transmission in non-educational settings [11–13]. Nonetheless, surging evidence suggests that adolescents face a comparable risk of infection to the general population and the age dependent variability in infection transmission should be considered when devising control strategies [14]. Hence, we examined children’s testing data during two consecutive academic years to estimate testing patterns, incidence rates, and percentage of positive SARS-CoV-2. The epidemiological parameters will be compared to adults’ rates through major COVID-19 waves, the rest of the academic year and school holidays.

Furthermore, there is limited evaluation of the timely implementation of physical distancing interventions, particularly national schools’ closures, and their impact on infection trends. Accordingly, we explored the application of the CDC indicators for community transmission namely: population incidence, and percentage of positive SARS-CoV-2 cases to identify data-driven alert thresholds that can be used to guide timely adjustments in public health response especially school attendance policies [15, 16], (refer to Supplementary files “SF3, SF4”).

## Materials and methods

Qatar has an integrated electronic health information system that captures all COVID-19 disease related data including all records of polymerase-chain-reaction (PCR) testing, and rapid antigen tests (RAT) conducted at all health care venues in Qatar. RAT started in December 2021 (21st week of 2021–2022 academic year). Tests were provided at no cost or at heavily subsidized rates [4]. All the laboratory COVID-19 testing was centralized and was conducted at Hamad Medical Corporation’s (HMC)-the main healthcare provider in Qatar- central laboratory using real-time reverse-transcription PCR (RT-qPCR) TaqPath COVID-19 Combo Kit [4]. We used the national database to identify all tests conducted between August 26th, 2020, and August 21st of 2022 in all age groups (≥ 5 years old). These databases are comprehensive, containing no missing data [5]. Demographic characteristics were extracted from the anonymized electronic medical records and no identifying information was collected.

Rapid antigen self-testing kits were available for purchase in pharmacies in 2022; however, the outcomes of home-based testing are neither reported nor documented in the national database [5].

### Data analysis

The study subjects (< 18 years old) were stratified into two age categories: (5–11) (12–17) to match the Primary and Preparatory/Secondary grade levels in Qatar, respectively. We estimated age groups testing rates, incidence rates, and positivity in comparison to adults (≥ 18 years old) during the two separate academic years. All the former epidemiological measures were expressed as number of cases per 100,000 population per week. Although subjects (< 18 years old) were accounted for in two consecutive years, our analysis primarily focused on test positivity rates on each test day without any further follow-up. We believe the potential bias introduced by this approach is minimal and will not have any significant impact on our findings.

We referred to the first school year as (2020–2021) or Y1 interchangeably, and we did the same for the second school year. We referred to the Alpha/Beta/Delta wave as (W1), and the Omicron wave as (W2) (refer to Supplementary files “SF1”, “SF2”).

Positivity rate was defined as the number of positive COVID-19 infections (identified by RT-PCR, or RAT) over the total number of all tests. The one-way analysis of variance test (ANOVA) was used to determine the statistically significant differences between different age categories in the testing rate, incidence, and positivity rates. The mean difference was considered significant at the 0.05 level (*p* ≤ 0.05). 7-days moving average was calculated to flatten anticipated variation in daily case numbers and tests’ positivity. Games-Howell test was used for post hoc analysis to determine the statistical significance of differences in group pairs means. Additionally, we examined the differences among age groups during Y1 for the following periods: School academic periods (weeks 1–16, weeks 39–45), holidays (weeks 17–20 & 46–53), and during COVID-19 W1 (weeks 23–38). During Y2, the winter break was extended for an extra 4 weeks as a response to the W2. We examined the difference in study academic periods (weeks 1–16, weeks 25–45), holidays (46–53), and during COVID-19 W2 (weeks 17–24).

For comparison, we used the CDC threshold for highest transmission of ≥ 200 new COVID-19 cases per 100,000 population in the last 7 days, and the percentage of positive RT-PCR tests ≥ 10% in the last 7 days. All statistical data analysis was conducted using STATA/MP 15.1.

## Results

A total of 5,063,405 laboratory tests were conducted in the school year (2020–2021), while 6,130,531 tests were conducted in the school year (2021–2022). In Y1, most tests, accounting for 89.6% (*n* = 4,537,604), were administered to adults. Similarly, in the Y2, only 13.7% (*n* = 840,634) of the tests were conducted on individuals under 18 years old. Males underwent testing more frequently than females. Rapid antigen testing (RAT) was introduced during Y2 with 84.5% of tests administered to adults, 6.9% to individuals aged 12–17 years, and 8.6% to those aged 5–11 years. The overall test positivity rates for the (2020–2021) and (2021–2022) school years were 5.8% and 8.1%, respectively. During the full study period, adolescents underwent the fewest tests in comparison to both adults and young children (refer to Table 1).

**Table 1:** Characteristics of persons tested and testing outcomes

	**Academic Year (2020–2021)** **n (%)**				**Academic Year (2021–2022)** **n (%)**			
**Age group**	**( ≥)18y**	**(12–17) y**	**(5–11) y**	**Total**	**( ≥)18y**	**(12–17) y**	**(5–11) y**	**Total**
Gender								
Female	1,197,230 (83.1)	98,124 (6.8)	145,517 (10.1)	1,440,871 (28.5)	1,694,293 (80.8)	156,611 (7.5)	247,087 (11.8)	2,097,991 (34.2)
Male	3,340,374 (92.2)	127,614 (3.5)	154,546 (4.3)	3,622,534 (71.5)	3,595,604 (89.2)	172,803 (4.3)	264,133 (6.6)	4,032,540 (67.8)
**Nationality**								
Non-Qatari	3,923,534 (92.1)	129,716 (3.0)	205,474 (4.8)	4,258,724 (84.1)	4,269,778 (88.9)	177,930 (3.7)	353,757 (7.4)	4,801,465 (78.3)
Qatari	61,4070 (76.3)	96,022 (11.9)	94,589 (11.8)	804,681 (16.0)	1,020,119 (76.8)	151,484 (11.4)	157,463 (11.8)	1,329,066 (21.7)
**Test type**								
PCR	4,537,604 (89.6)	225,738 (4.5)	300,063 (5.9)	5,063,405 (100.0)	4,025,380 (86.9)	226,620 (4.9)	382,508 (8.3)	4,634,508 (75.6)
RAT	0 (0.0)	0 (0.0)	0 (0.0)	0 (0.0)	1,264,517 (84.5)	102,794 (6.9)	128,712 (8.6)	1,496,023 (24.4)
** Test outcome**								
COVID-19 Positive	263,852 (89.5)	13,599 (4.6)	17,461 (5.9)	294,912 (5.8)	418,390 (84.2)	32,454 (6.5)	46,279 (9.3)	497,123 (8.1)
COVID-19 Negative	4,273,752 (89.6)	212,139 (4.4)	282,602 (5.9)	4,768,493 (94.2)	4,871,507 (86.5)	296,960 (5.3)	464,941 (8.3)	5,633,408 (92.0)
**Total**	**4,537,604 (89.6)**	**225,738 (4.5)**	**300,063 (5.9)**	**5,063,405**	**5,289,897 (86.3)**	**329,414 (5.4)**	**511,220 (8.3)**	**6,130,531**

### Testing capacity

**Fig. 1:**
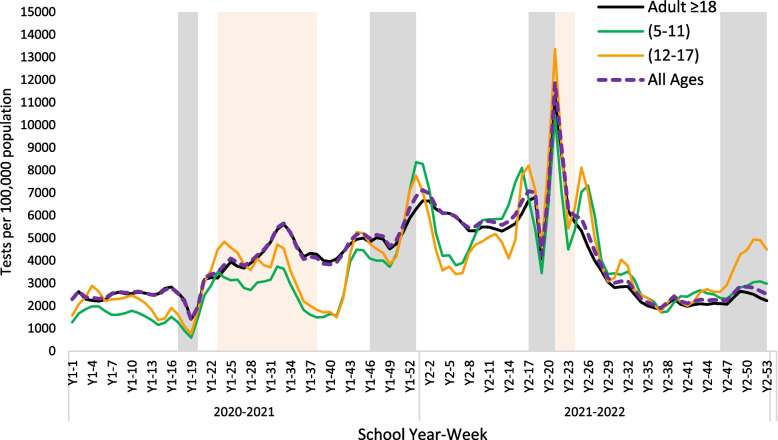
Number of weekly COVID-19 tests during school year (2020–2021) and (2021–2022) for different age groups. School holidays are marked in gray, and COVID-19 waves are marked in light pink. A 7-day running average was calculated

During Y1 academic period, there was a clear correlation between testing rates and the age groups of the study participants. The mean testing rates per 100,000 population per week were 3,057 for adults, 2,403 for individuals aged 12–17 years and 1,978 for those aged 5–11 years, with a statistically significant difference observed (*p* < 0.05). During W1, the testing capacity increased towards week 21 (end of January 2021). By the end of March 2021, more tests were conducted at a rate of 4,297 for adults, 3,763 for those aged 12–17 years old, and 2,904 for 5–11 years old children, *p* < 0.000, (refer to Table 2). The testing trend continued until around Week 34 (beginning of April) when the test rates for youth began to decrease, coinciding with the closure of schools and the transition to 100% online classes. Figure 1 shows that the testing rates increased by the end of the schools’ summer vacation as Qatar’s residents (90% expats) were required to obtain a Covid-19 negative certificate by taking a PCR test after travel.

**Table 2:** Mean rates per 100,000 population per week

	**Academic Year (2020–2021)**				**Academic Year (2021–2022)**			
	Adult ≥ 18	(12–17)	(5–11)	*p* value	Adult ≥ 18	(12–17)	(5–11)	*p* value
**Testing Rate**								
COVID-19 wave	4,297	3,763	2,904	*P* = 0.000	6,723	7,872	6,330	*P* = 0.015
School academic period	3,057	2,403	1,978	*P* = 0.035	4,075	3,985	4,409	*P* = 0.000
School Holidays	4,061	3,786	3,552	*p* = 0.230	3,356	4,734	3,511	*P* = 0.032
**Incidence**								
COVID-19 wave	466	424	325	*P* = 0.047	1,771	2,238	1,804	*P* = 0.000
School academic period	116	102	83	*P* < 0.001	79	139	170	*P* = 0.031
School Holidays	76	64	54	*P* = 0.202	43	71	98	*P* = 0.072
**Positivity**								
COVID-19 wave	10.2%	11.7%	11.5%	*P* = 0.002	22.0%	25.0%	25.6%	*P* = 0.000
School period	4.0%	4.7%	4.8%	*P* = 0.025	4.4%	6.3%	6.3%	*P* = 0.015
School Holidays	2.2%	2.1%	2.3%	*P* = 0.122	7.0%	7.0%	7.4%	*P* = 0.518

Analysis Of Variance (ANOVA) test was used for comparing the means. The mean difference was considered significant at the 0.05 level (*p* < 0.05)

Games-Howell Post hoc test was used for post hoc analysis to determine the statistical significance of differences in group pairs means

COVID-19 wave in Y1: Alpha/Beta/Delta variants wave (Refer to Supplementary files “SF1”, “SF2”)

COVID-19 wave in Y2: Omicron variant wave (Refer to Supplementary files “SF1”, “SF2”

Looking at the consecutive academic year (2021–2022) we notice that there was a great increase in the testing rates in general. During the academic period, the mean testing rate was the highest among the younger age group at 4,409 per week per 100,000 population, compared to 4,075 for adults, 3,985 for (12–17 years old), *p* < 0.000. During W2 the testing rates was the highest in 12–17 years old with a mean of 7,872 tests per 100,000 population compared to 6,723 in adults, and 6,330 tests in the younger age group *P* < 0.05. This reflects the increase in testing as younger age cohorts were required to show a negative test result (home based RAT) to attend schools especially unvaccinated children (refer to Fig. 1, Supplementary files “SF3”). Despite home testing not being documented in the national data repository, individuals who tested positive were directed by the Ministry of Public Health (MOPH) to visit the nearest health center. There, they would likely undergo testing again, adding a second layer of asymptomatic testing for youth.

### Incidence

**Fig. 2:**
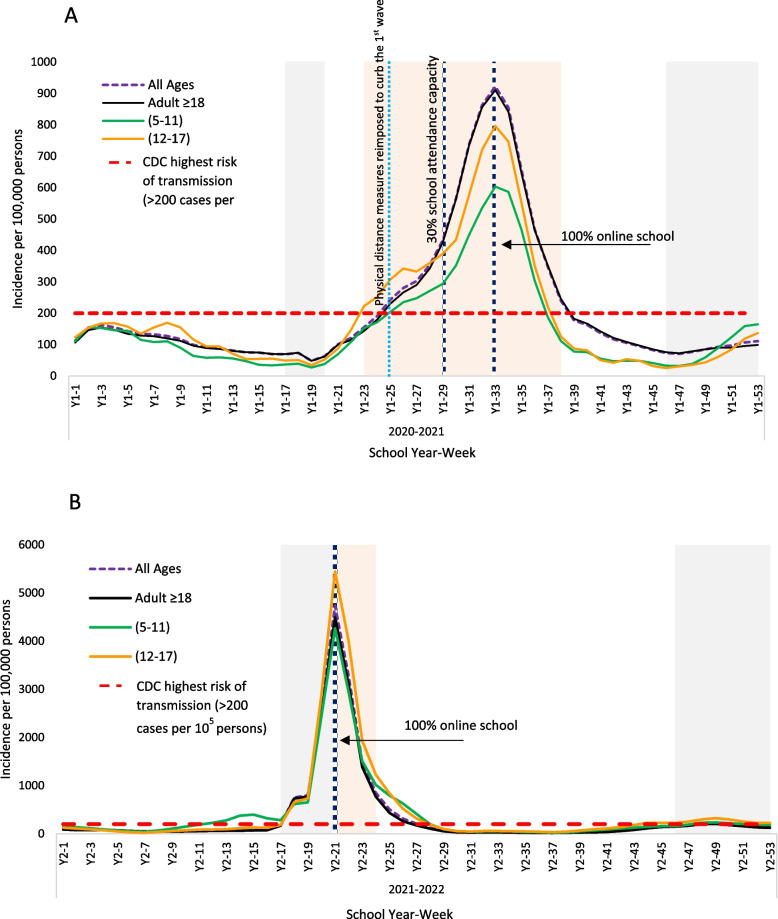
Cases per 100,000 population during school year (2020–2021 [**A**]) and (2021–2022 [**B**]) for different age groups. School holidays are marked in gray, and COVID-19 waves are marked in light pink. A 7-day running average was calculated. The number of weekly cases was compared to the CDC indicator for risk of transmission in schools

During Y1 academic period, the mean incidence rates per 100,000 population per week were as follows: 116 for adults, 102 for individuals aged 2–17, and 83 for those aged 5–11, respectively (*p* < 0.001; see Table 2 and Fig. 2). When examining the differences among age groups in W1, the mean incidence rate associated directly with the age group of the subjects at 466 for adults, 424 for individuals aged 12–17, and 325 for those aged 5–11 (*p* < 0.05). The rates were the lowest during school holidays at 76 for adults, 64 for individuals aged 2–17, and 54 for those aged 5–11, respectively. However, this difference was not statistically significant (*p* > 0.05).

By the end of January 2021, there was a sharp increase in incidence among all age groups, surpassing the CDC threshold for COVID-19 community transmission levels (see Fig. 2A). Schools transitioned to 30% on-campus attendance in March, and by the first week of April, all on-campus attendance was halted. Throughout this period, the rate continued to rise across all age groups, reaching 642 for adults, 539 for individuals aged 12–17, and 412 for those aged 5–11, respectively. Following April, there was a noticeable decline in the number of weekly cases. During this period, adults continued to exhibit the highest rates at 321 cases per 100,000 population, compared to 232 among individuals aged 12–17, and 195 among those aged 5–11 years old.

At the beginning of Y2's academic period (refer to Fig. 2B), the incidence rates among all age groups consistently remained below the CDC threshold. The mean rates were 79, 139, and 170 cases per 100,000 population per week for adults, individuals aged 12–17, and those aged 5–11, respectively (*p* < 0.05). However, the rates among children aged 5–11 exceeded the CDC threshold in the first week of November (week 12), reaching 226 cases per 100,000 persons per week, compared to 90 among adolescents and 59 among adults. This trend persisted until around mid-December (week 17).

There was a significant increase in levels around weeks 18–20. Children in both age categories experienced higher mean incidence rates, reaching 2238 cases among 12–17 year-olds and 1804 among 5–11 year-olds. Adults had a lower incidence rate at 1771 per 100,000 persons per week. Schools transitioned to 100% online learning after the winter break (week 21) until the end of January. Subsequently, schools shifted to 100% face-to-face attendance at week 25. During this period, there was a notable decrease in incidence until the end of the school year, reaching 43 among adults, 71 among 12–17 year-olds, and 98 among 5–11 year-olds, respectively (*p* < 0.05) (see Fig. 2B).

### Positivity

**Fig. 3:**
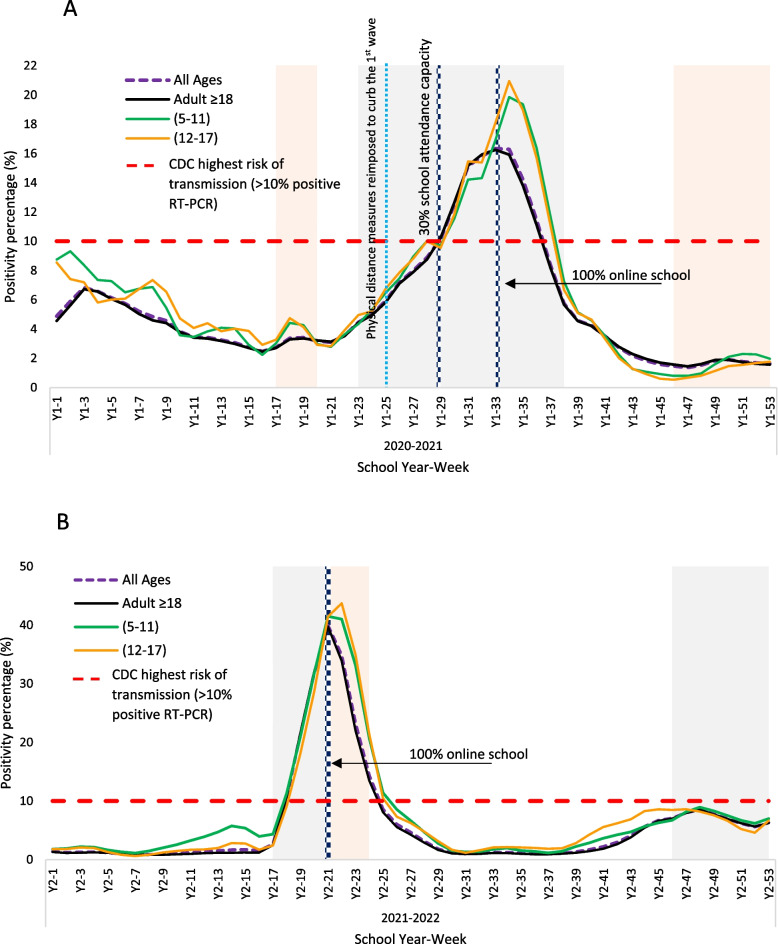
Weekly positivity rate for COVID-19 during school year (2020-2021 [2:A]) and (2021-2022 [2:B]) for different age groups. School holidays are marked in gray, and COVID-19 waves are marked in light pink. A 7-day running average was calculated. The number of weekly cases was compared to the CDC indicator for risk of transmission in schools

At the start of the study period in Year 2020–2021, tests conducted on adults revealed a mean weekly positivity rate of 4%, while the rates were slightly higher at 4.7% for the 12–17 age group and 4.8% for the 5–11 age group (*p* > 0.05). in January 2021, following the winter break, positivity rates of RT-PCR tests steadily increased, surpassing the CDC threshold of 10% by week 28 (the third week of February). During W1, the rates continued to increase at a mean positivity rates of 12.7% for adults, 16% for individuals aged 12–17, and 16% for those aged 5–11 (see Table 2 and Fig. 3:A). This trend could be attributed to the higher testing rates among adults, with 4856 tests conducted per 100,000 persons compared to 3139 among individuals aged 12–17 and 2543 among those aged 5–11, as schools transitioned to 100% online attendance (*p* < 0.05).

In Y2’s academic period, The positivity rates exceeded the CDC threshold around week 18, with test positivity rates reaching 11.3% among individuals aged 5–11, 10.7% among adults, and 9.1% among those aged 12–17. During W2, the mean positivity rates were the highest among children aged 5–11 at 25.6%, compared to 25% for individuals aged 12–17, and 22% for adults (*p* < 0.001). This suggests higher community transmission among younger age groups and indicates the need for increased testing frequency for children. Subsequently, there was a significant decrease in levels over the following months (weeks 25–45) (see Fig. 3:B), with children aged 5–11 exhibiting the lowest mean positivity rate at 3.7%, compared to 4.4% in individuals aged 12–17 and 4.7% in adults (*p* < 0.001).

## Discussion

In general, trends in incidence and positivity rates across all age subgroups of children mirrored those observed in adults over the duration of the study. Using the CDC indicator for new number of cases as a benchmark, we noticed that in January 2021, the incidence of COVID-19 among adolescents surpassed that among adults. It preceded the increase among adults by around 2 weeks (weeks 23–25). This trend continued until the begging of March. One plausible explanation could be the initiation of the COVID-19 national vaccination program in December 2020. This program was structured to prioritize frontline healthcare workers, individuals with severe or multiple chronic conditions, and those aged 70 years and older [17]. Furthermore, Qatar, during the same period, started gradual lifting of social restrictions and increased the educational and workplace capacity. The observed difference between young children and adolescents (see Fig. 2) could be attributed to the limited opportunities for exposure and testing among children under 12 years old. For a significant portion of Y1, children under 12 were barred from entering shopping malls or enclosed spaces [18].

In Year 2, incidence rates among children aged 5 to 11 began to surpass those of other age groups around mid-October (week 9), coinciding with schools returning to full attendance capacity on October 3rd, 2021.The trend continued with incidence levels among young children passing the CDC incidence rate threshold in the first week of November (week12), followed by the adult and adolescent groups in the second week of December (week 18). The lower incidence among adults during this period cannot be attributed to vaccination accessibility, as the Ministry of Public Health (MOPH) integrated adolescents aged 12 to 15 into the vaccination campaign in May 2021.

Schneiderman and colleagues reported similar findings to our results in a series of studies in USA [19, 20]. They hypothesized that adolescents had greater contact rates compared to adults, and that older adults, perceiving themselves as more vulnerable, were more inclined to adhere to masking guidelines or social distancing measures [19, 20]. Additional research in in Shenzhen, China [21, 22] indicates that children face a comparable risk of infection to that of the general population, with the rate of infection among young children reported at 7.4%, in comparison to the population average of 7.9%. Our research asserts that children can contribute significantly to elevated infection rates, particularly following the easing of control measures. The potential for high transmission among youth and adolescents should be considered in transmission control and developing physical distancing strategies, especially schools closure decisions.

Moreover, in our analysis we assessed the timing of school closures implemented by public health authorities in relation to infection trends. The efficacy of school closures in controlling SARS-CoV-2 transmission is a topic of ongoing debate, and it remains an area for further research. Initial evidence indicated that school closures had only minimal effectiveness in controlling infections [23]. However, another study indicated that implementing school closures earlier during periods of lower COVID-19 cumulative incidence was associated with a reduction of 128.7 cases per 100,000 population [24]. Yang, W et al. noted that reducing contact rates, primarily through school closures and stay-at-home orders, contributed to the most significant transmission reduction among the population of New York City [25].

During the surge in coronavirus cases in the Y1, social restrictions were reinstated in February 2021 to mitigate the wave, while schools maintained operations with a 30% on-campus capacity. However, it was not until April 4th, 2021 that schools transitioned to remote online learning for all students, more than 60 days after reaching the CDC thresholds and implementing initial social distancing measures. Afterwards, on April 9th, public health officials reinstated stricter preventive measures, prohibiting indoor social gatherings as well as gatherings in parks and beaches (Refer to the Supplementary file “SF3”).

Considering the context of relatively higher community transmission in Qatar [2], the emergence of the novel Delta variant (B.1.617.2) and the lower vaccination coverage at the time, we argue that implementing school closures earlier, between weeks 25 and 29, rather than in April (week 33), would have been more effective in mitigating transmission and flattening the epidemic curve [26].

We hypothesize that during Year 2, the implementation of physical restriction measures, particularly reduced school attendance, should have commenced one month earlier, in week 12, rather than in week 18 when the schools began their winter break. However, it is essential to acknowledge that public health officials based their decisions within the context of the reduced viral pathogenicity of the Omicron variant [27] and the higher population immunity compared to earlier stages of the pandemic. By December 2021, approximately 86% of Qatar's population had received two doses of the COVID-19 vaccines [28].

Research indicates that implementing physical distancing measures concurrently as a “basket of measures” [29], without delay, would decrease the scale of the epidemic, postpone its peak, and prevent healthcare systems from becoming overwhelmed [28]. Our findings offer insights that can guide control efforts for other (re)emerging infections in the future.

A limitation of our study is the potential underestimation of infection rates among younger age groups during the study period. Our analysis demonstrates that children, particularly adolescents, were tested less frequently than adults. Throughout the study period, only 10%-14% of all tests were conducted on individuals under 18 years old. This might’ve resulted in underreporting of cases within this age group and a corresponding underestimation of incidence rates. Additionally, isolating the impact of school closures from the broader spectrum of public health measures is challenging. In Qatar, school closures typically coincided with other pandemic control measures, making it difficult to ascertain its individual effect.

## Conclusion

The COVID-19 pandemic has posed considerable challenges for healthcare systems and policymakers. Our study's results, indicating higher incidence of COVID-19 among school age adolescents and young individuals, support the theories suggesting a greater potential for transmissibility in these age cohorts. Utilizing data-driven indicators and thresholds to monitor COVID-19 community levels is essential for informing decision-making regarding health strategies and transmission mitigation. Such approaches also facilitate the timely implementation of infection control measures in future pandemics.

### Supplementary Information

Supplementary Material 1.

## Data Availability

The data supporting the findings of this study are available within the article [and/or] its Supplementary materials.

## Abbreviations

COVID-19: Corona Virus Disease 2019
CDC: Center for Disease Control and Prevention
MOEHE: Ministry of Education and Higher Education
PHCC: Primary Health Care Corporation
IRB: Institutional Review Board
W1: Alpha/Beta/Delta variants wave
W2: Omicron variant Wave

## References

[1] Ministry of Public Health, Qatar (MOPH). COVID-19 home. 2020. Retrieved from: https://covid19.moph.gov.qa/EN/Pages/default.aspx.

[2] WHO. East Mediterranean Regional Office COVID-19 Dashboard. (2024). Retrieved from: https://app.powerbi.com/view?r=eyJrIjoiN2ExNWI3ZGQtZDk3My00YzE2LWFjYmQtNGMwZjk0OWQ1MjFhIiwidCI6ImY2MTBjMGI3LWJkMjQtNGIzOS04MTBiLTNkYzI4MGFmYjU5MCIsImMiOjh9.

[3] Nakhaeizadeh M, Eybpoosh S, Jahani Y (2022). Impact of non-pharmaceutical interventions on the control of COVID-19 in Iran: a mathematical modeling study. Int J Health Policy Manag.

[4] AlNuaimi AA, Chemaitelly H, Semaan S (2023). All-cause and COVID-19 mortality in Qatar during the COVID-19 pandemic. BMJ Glob Health.

[5] Abu-Raddad LJ, Chemaitelly H, Ayoub HH (2021). Characterizing the Qatar advanced-phase SARS-CoV-2 epidemic. Sci Rep.

[6] Ministry of Education and Higher Education (MOEHE). The general orientation of the ministry in the next educational stage for public and private schools. 2020. Retrieved from: https://www.edu.gov.qa/ar/mediacenter/Pages/MediaCenter/NewsDetails.aspx?itemid=194.

[7] Royal Society DELVE Initiative. Balancing the Risks of Pupils Returning to Schools. 2020. Retrieved from: https://rs-delve.github.io/reports/2020/07/24/balancing-the-risk-of-pupils-returning-to-schools.html#1-background.

[8] Chemaitelly H, Tang P, Coyle P (2023). Protection against reinfection with the omicron BA.2.75 subvariant. N Engl J Med.

[9] Tang P, Hasan MR, Chemaitelly H (2021). BNT162b2 and mRNA-1273 COVID-19 vaccine effectiveness against the SARS-CoV-2 delta variant in Qatar. Nat Med.

[10] Chemaitelly H, Tang P, Hasan MR (2021). Waning of BNT162b2 vaccine protection against SARS-CoV-2 infection in Qatar. N Engl J Med.

[11] Macartney, K, Quinn, HE, Pillsbury, AJ, Koirala, A, Deng, L, Winkler, N, Katelaris, AL, O'Sullivan, M, Dalton, C, Wood, N, & NSW COVID-19 Schools Study Team. (2020). Transmission of SARS-CoV-2 in Australian educational settings: a prospective cohort study. The Lancet. Child & adolescent health, S2352-4642(20)30251-0. Advance online publication. Retrieved from: 10.1016/S2352-4642(20)30251-0. 10.1016/S2352-4642(20)30251-0 PMC7398658 32758454

[12] Yung CF, Kam KQ, Nadua KD, Chong CY, Tan N, Li J, Lee KP, Chan YH, Thoon KC, Ng KC (2021). Novel coronavirus 2019 transmission risk in educational settings. Clin Infect Dis.

[13] Ismail SA, Saliba V, Lopez Bernal J, Ramsay ME, Ladhani SN (2021). SARS-CoV-2 infection and transmission in educational settings: a prospective, cross-sectional analysis of infection clusters and outbreaks in England. Lancet Infect Dis.

[14] Mader S, Rüttenauer T (2022). The effects of non-pharmaceutical interventions on COVID-19 mortality: a generalized synthetic control approach across 169 countries [published correction appears in Front Public Health. 2023 Mar 27;11:1186935]. Front Public Health.

[15] CDC. Indicators for Dynamic School Decision-Making. 2020. Retrieved from:https://www.cdc.gov/coronavirus/2019-ncov/community/schools-childcare/indicators.html#thresholds.

[16] CDC. Science Brief: Transmission of SARS-CoV-2 in K-12 Schools and Early Care and Education Programs – Updated. 2021. Retrieved from: https://www.cdc.gov/coronavirus/2019-ncov/science/science-briefs/transmission_k_12_schools.html. 34009772

[17] Albayat S, Almaslamani M, Alromaihi H (2023). Key lessons from COVID-19: a narrative review describing Qatar's multifactorial approach in executing a vaccination campaign. Vaccines (Basel).

[18] Qatar Tribune. Children below 12 years not allowed to enter malls. 2022. Retrieved from: https://www.qatar-tribune.com/article/8321/TopNews/Children-below-12-years-not-allowed-to-enter-malls.

[19] Schneiderman M, Rumain B, Kaganovskiy L, Geliebter A (2022). Incidence and relative risk of COVID-19 in adolescents and youth compared with older adults in 19 US States, Fall 2020. JAMA Netw Open.

[20] Rumain B, Schneiderman M, Geliebter A (2021). Prevalence of COVID-19 in adolescents and youth compared with older adults in states experiencing surges. PLoS One.

[21] Aguas R, White L, Hupert N (2020). Modelling the COVID-19 pandemic in context: an international participatory approach [published correction appears in BMJ Glob Health. 2021 Feb;6(2):]. Glob Health.

[22] Bi Q, Wu Y, Mei S (2020). Epidemiology and transmission of COVID-19 in 391 cases and 1286 of their close contacts in Shenzhen, China: a retrospective cohort study [published correction appears in Lancet Infect Dis. 2020 Jul;20(7):e148].. Lancet Infect Dis.

[23] Banholzer N, van Weenen E, Lison A (2021). Estimating the effects of non-pharmaceutical interventions on the number of new infections with COVID-19 during the first epidemic wave. PLoS One..

[24] Auger KA, Shah SS, Richardson T (2020). Association between statewide school closure and COVID-19 incidence and mortality in the US. JAMA.

[25] Yang W, Shaff J, Shaman J (2021). Effectiveness of non-pharmaceutical interventions to contain COVID-19: a case study of the 2020 spring pandemic wave in New York City. J R Soc Interface.

[26] Sharifi H, Jahani Y, Mirzazadeh A (2022). Estimating COVID-19-related infections, deaths, and hospitalizations in iran under different physical distancing and isolation scenarios. Int J Health Policy Manag.

[27] Sigal A (2022). Milder disease with Omicron: is it the virus or the pre-existing immunity?. Nat Rev Immunol.

[28] Ministry of Public Health, Qatar (MOPH). National Covid-19 Vaccination Program Data. 2021. Retrieved from: https://covid19.moph.gov.qa/EN/Pages/Vaccination-Program-Data.aspx

[29] Summan A, Nandi A (2022). Timing of non-pharmaceutical interventions to mitigate COVID-19 transmission and their effects on mobility: a cross-country analysis. Eur J Health Econ.

