# Supplementary material for: COVID-19 testing, incidence, and positivity trends among school age children during the academic years 2020–2022 in the State of Qatar: special focus on using CDC indicators for community transmission to evaluate school attendance policies and public health response
Source: BMC Pediatr. 2024 May 30;24:374. doi: 10.1186/s12887-024-04833-9 (PMC11137921; doi:10.1186/s12887-024-04833-9)
Supplement: Supplementary file 1 — Supplementary Material 1. [file 12887_2024_4833_MOESM1_ESM.docx]

| **SF (1): Academic Week and Corresponding Dates** | | |
| --- | --- | --- |
| **Academic Week** | **2020-2021 (Y1)** | **2021-2022 (Y2)** |
| 1 | 26/08/2020 | 22/08/2021 |
| 2 | 30/08/2020 | 29/08/2021 |
| 3 | 06/09/2020 | 05/09/2021 |
| 4 | 13/09/2020 | 12/09/2021 |
| 5 | 20/09/2020 | 19/09/2021 |
| 6 | 27/09/2020 | 26/09/2021 |
| 7 | 04/10/2020 | 03/10/2021 |
| 8 | 11/10/2020 | 10/10/2021 |
| 9 | 18/10/2020 | 17/10/2021 |
| 10 | 25/10/2020 | 24/10/2021 |
| 11 | 01/11/2020 | 31/10/2021 |
| 12 | 08/11/2020 | 07/11/2021 |
| 13 | 15/11/2020 | 14/11/2021 |
| 14 | 22/11/2020 | 21/11/2021 |
| 15 | 29/11/2020 | 28/11/2021 |
| 16 | 06/12/2020 | 05/12/2021 |
| 17 | 13/12/2020 | 12/12/2021 |
| 18 | 20/12/2020 | 19/12/2021 |
| 19 | 27/12/2020 | 26/12/2021 |
| 20 | 01/01/2021 | 02/01/2022 |
| 21 | 03/01/2021 | 09/01/2022 |
| 22 | 10/01/2021 | 16/01/2022 |
| 23 | 17/01/2021 | 23/01/2022 |
| 24 | 24/01/2021 | 30/01/2022 |
| 25 | 31/01/2021 | 06/02/2022 |
| 26 | 07/02/2021 | 13/02/2022 |
| 27 | 14/02/2021 | 20/02/2022 |
| 28 | 21/02/2021 | 27/02/2022 |
| 29 | 28/02/2021 | 06/03/2022 |
| 30 | 07/03/2021 | 13/03/2022 |
| 31 | 14/03/2021 | 20/03/2022 |
| 32 | 21/03/2021 | 27/03/2022 |
| 33 | 28/03/2021 | 03/04/2022 |
| 34 | 04/04/2021 | 10/04/2022 |
| 35 | 11/04/2021 | 17/04/2022 |
| 36 | 18/04/2021 | 24/04/2022 |
| 37 | 25/04/2021 | 01/05/2022 |
| 38 | 02/05/2021 | 08/05/2022 |
| 39 | 09/05/2021 | 15/05/2022 |
| 40 | 16/05/2021 | 22/05/2022 |
| 41 | 23/05/2021 | 29/05/2022 |
| 42 | 30/05/2021 | 05/06/2022 |
| 43 | 06/06/2021 | 12/06/2022 |
| 44 | 13/06/2021 | 19/06/2022 |
| 45 | 20/06/2021 | 26/06/2022 |
| 46 | 27/06/2021 | 03/07/2022 |
| 47 | 04/07/2021 | 10/07/2022 |
| 48 | 11/07/2021 | 17/07/2022 |
| 49 | 18/07/2021 | 24/07/2022 |
| 50 | 25/07/2021 | 31/07/2022 |
| 51 | 01/08/2021 | 07/08/2022 |
| 52 | 08/08/2021 | 14/08/2022 |
| 53 | 15/08/2021 | 21/08/2022 |

**SF(2): SARS-CoV-2 epidemic waves and incidence rate per 100,000 population per week between August 26, 2020 and August 21, 2022**.


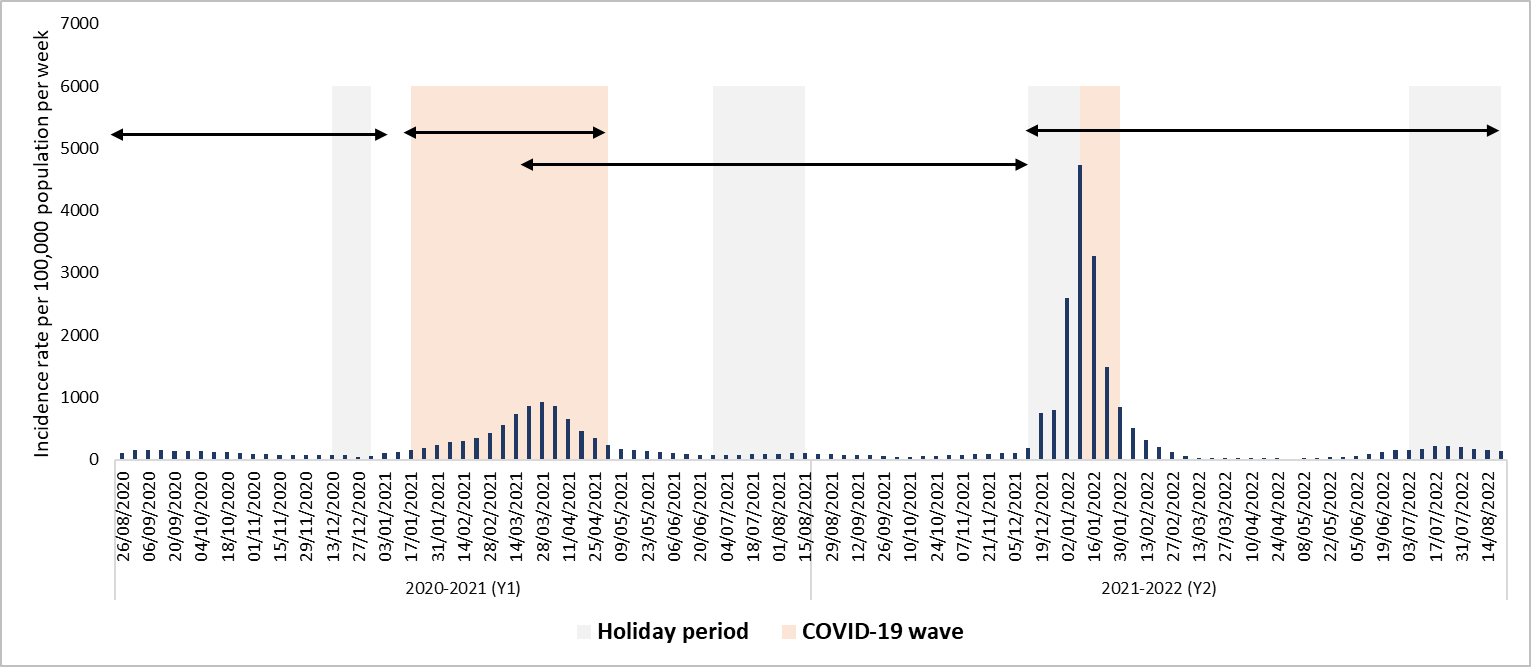


Index virus

Alpha/Beta (W1) variants

Delta variant

Omicron variant (W2)

| **SF (3): Physical distancing preventive measures with corresponding dates** | | | | |
| --- | --- | --- | --- | --- |
| **Dat** | **Preventive measure** | **Academic Week** | **Academic Year** | **Reference** |
| **01-Sep-20** | Blended learning- online or in school, maximum 50% class strength | 1 | Y1 | <https://hukoomi.gov.qa/en/news/moehe-considers-applying-blended-learning-for-school-year-2020-2021> |
| **20-Sep-20** | blended learning system, maintaining 30% occupancy rate, parents an option between continuing in the blended learning path or switching to completely distance learning to control attendance | 5 | Y1 | <https://hukoomi.gov.qa/en/news/moehe-decides-to-give-parents-option-between-blended-or-distance-learning> |
| **01-Nov-20** | compulsory attendance, 42% percent of the school capacity, weekly rotational attendance schedule | 11 | Y1 | <https://hukoomi.gov.qa/en/news/moehe-approves-new-system-for-student-attendance-in-public-and-private-schools> |
| **03-Jan-21** | School reopens after winter break, 50% rotational on-site school attendance. | 20 | Y1 | <https://hukoomi.gov.qa/en/news/moehe-decides-to-continue-blended-learning-and-increase-attendance-rate-up-to-50-during-second-term> |
| **21-Mar-21** | Reduced attendance 30% | 32 | Y1 | <https://hukoomi.gov.qa/en/news/moehe-student-attendance-in-all-schools-to-be-30-starting-from-Sunday> |
| **21-Mar-21** | Mandatory vaccination for employees | 32 | Y1 | <https://hukoomi.gov.qa/en/news/moehe-preventing-employees-from-entering-schools-except-on-the-condition-of-vaccination-or-examination> |
| **04-Apr-21** | 100% Online classes | 34 | Y1 | <https://www.edu.gov.qa/en/MediaCenter/Pages/MediaCenter/NewsDetails.aspx?itemid=287> |
| **16-May-21** | Vaccination campaign for 12-15 years started | 40 | Y1 | <https://hukoomi.gov.qa/en/news/moph-opens-online-registration-for-children-aged-12-to-15-in-covid-19-vaccine> |
| **01-Sep-21** | New academic year starts, a blended education system, with rotation 50% attendance on campus | 1 | Y2 | <https://hukoomi.gov.qa/en/news/moehe-decides-to-adopt-blended-education-in-all-schools-for-2021-2022-with-50-attendance> |
| **03-Oct-21** | Full capacity, 100% attendance, with masks | 1 | Y2 | <https://hukoomi.gov.qa/en/news/mmoehe-announces-the-return-of-study-with-full-attendance> |
| **01-Jan-22** | Extended holidays due to Omicron wave | 20 | Y2 | <https://hukoomi.gov.qa/en/news/moehe-decides-to-continue-distance-learning-system-until-january-27-2022> |
| **30-Jan-22** | School reopens with 100% class strength, to Conduct a rapid antigen test on a weekly basis at home for all students | 25 | Y2 | <https://hukoomi.gov.qa/en/news/back-to-school-with-full-attendance-from-Sunday> |
| **31-Jan-22** | Vaccination campaign for 5-11 years started | 25 | Y2 | <https://hukoomi.gov.qa/en/news/moph-approves-pfizer-biontech-covid-19-vaccines-for-children-aged-5-to-11-years> |
| **03-Apr-22** | Mask not mandatory, optional for students who wish to do so. Continuing weekly rapid antigen test on a weekly basis for unvaccinated and non-recovered students | 34 | Y2 | <https://www.edu.gov.qa/en/MediaCenter/Pages/MediaCenter/NewsDetails.aspx?itemid=457> |

**SF (4): Centers for Disease Control and Prevention indicators for COVID-19 community transmission**


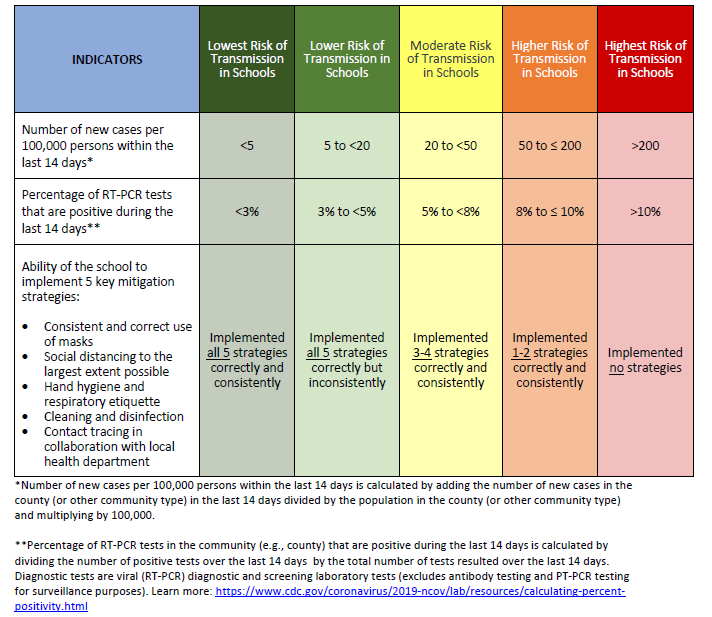


**Reference:** CDC. (2020). CDC Releases Indicators for Dynamic School Decision-Making Infographic

<https://www.cdc.gov/media/releases/2020/p0915-dynamic-school-decision-making-infographic.html>
